# The Psychosocial Predictors and Day-Level Correlates of Substance Use Among Participants Recruited via an Online Crowdsourcing Platform in the United States: Daily Diary Study

**DOI:** 10.2196/23872

**Published:** 2021-04-27

**Authors:** Jennifer Payaal Jain, Claudine Offer, Christopher Rowe, Caitlin Turner, Carol Dawson-Rose, Thomas Hoffmann, Glenn-Milo Santos

**Affiliations:** 1 San Francisco Department of Public Health San Francisco, CA United States; 2 Division of Epidemiology School of Public Health University of California, Berkeley Berkeley, CA United States; 3 Department of Epidemiology and Biostatistics School of Medicine University of California, San Francisco San Francisco, CA United States

**Keywords:** Amazon Mechanical Turk, stimulant use, alcohol use, craving, depression, affect, self-esteem, men who have sex with men

## Abstract

**Background:**

Alcohol consumption and stimulant use are major public health problems and contribute to morbidity and mortality in the United States. To inform interventions for substance use, there is a need to identify the day-level correlates of substance use by collecting repeated measures data in one’s natural environment. There is also a need to use crowdsourcing platforms like Amazon Mechanical Turk (MTurk) to efficiently engage larger populations of people who use alcohol and stimulants in research.

**Objective:**

We aimed to (1) utilize daily diaries to examine the temporal relationship between day-level cravings for alcohol and stimulant/substance use (ie, heavy drinking or any drug use) in a given day over 14 days and (2) assess whether depression, negative affect, and self-esteem measured at baseline predict substance use in a given day over 14 days among people who use alcohol and/or stimulants in the United States.

**Methods:**

Individuals aged ≥18 years in the United States, who reported alcohol or stimulant (ie, cocaine, crack cocaine, and methamphetamine) use in the past year, were recruited using MTurk between March 26 and April 13, 2018. Eligible participants completed a baseline survey and 14 daily surveys online. The baseline survey assessed sociodemographics and psychosocial (ie, depression, affect, self-esteem, and stress) factors. Daily surveys assessed substance use and cravings for alcohol and stimulants. Four multivariable random-intercept logistic regression models were built to examine psychosocial constructs separately along with other significant predictors from bivariate analyses while controlling for age and education.

**Results:**

Among a total of 272 participants, 220 were White, 201 were male, and 134 were men who have sex with men (MSM). The mean age was 36.1 years (SD 10.5). At baseline, 173 participants engaged in any current or past hazardous alcohol consumption, 31 reported using cocaine, 19 reported using methamphetamine, 8 reported using crack cocaine, and 104 reported any noninjection or injection drug use in the past 6 months. Factors independently associated with substance use were depression (adjusted odds ratio [aOR] 1.11, 95% CI 1.02-1.21; *P*=.01), negative affect (aOR 1.08, 95% CI 1.01-1.16; *P*=.01), lower levels of self-esteem (aOR 0.90, 95% CI 0.82-0.98; *P*=.02), and cravings for alcohol (aOR 1.02, 95% CI 1.01-1.03; *P*<.001) and stimulants (aOR 1.03, 95% CI 1.01-1.04; *P*=.01). MSM had higher odds of engaging in substance use in all models (model 1: aOR 4.90, 95% CI 1.28-18.70; *P*=.02; model 2: aOR 5.47, 95% CI 1.43-20.87; *P*=.01; model 3: aOR 5.99, 95% CI 1.55-23.13; *P*=.009; and model 4: aOR 4.94, 95% CI 1.29-18.84; *P*=.01).

**Conclusions:**

Interventions for substance use should utilize evidenced-based approaches to reduce depression, negative affect, and cravings; increase self-esteem; and engage MSM. Interventions may also consider leveraging technology-based approaches to reduce substance use among populations who use crowdsourcing platforms.

## Introduction

Alcohol consumption and drug use are major public health problems and contribute to a substantial amount of morbidity and mortality among adults in the United States [1-3]. Both psychosocial stressors and biobehavioral features play key roles in drug and alcohol use behaviors [4-6]. For instance, depression [7], negative affect (eg, guilt and shame) [8,9], stress [10,11], and low self-esteem [12,13] are known to characterize patterns of substance use. Further, craving, which can be described as an urge or desire to use drugs or alcohol, is a key biobehavioral aspect of substance use disorder (SUD) [6,14] and has been linked to drug and alcohol use in several studies [6,15,16]. However, understanding how these psychosocial factors and biobehavioral features influence substance use on a day-to-day basis remains understudied, especially among populations who use crowdsourcing platforms.

The relationship between craving and substance use is difficult to measure with accuracy because of the transient nature of craving [17]. Further, substance use can be episodic and is often shaped by mood and context (eg, social setting) [18]. Therefore, in order to more accurately capture the daily patterns associated with substance use, methodological approaches that overcome these challenges should be leveraged. The daily dairy method [18-20] offers a promising opportunity to identify the day-level correlates of substance use by collecting repeated measures over time in one’s natural environment, thereby taking into account within-person variation related to mood and context [17,18,21].

Substance use research generally relies on traditional recruitment methods (eg, targeted sampling and respondent driven sampling), which are expensive and time consuming [22,23]. Additionally, people who use alcohol or other drugs are often difficult to retain in research, which can result in small sample sizes and limited generalizability [23]. Amazon Mechanical Turk (MTurk) is an innovative crowdsourcing platform that can be used to overcome these limitations by efficiently recruiting and engaging larger populations of people who use alcohol or other drugs in research using the internet [23]. Although MTurk has been used more widely over the past decade, it is still underutilized, and its use may improve the scientific rigor of substance use research by overcoming the limitations noted above. In addition, MTurk may reduce underreporting bias by enabling participants to report on sensitive behaviors in private settings [23]. More research, which leverages MTurk and the daily diary method together, is needed to engage larger populations of people who use alcohol or other drugs in research and to identify the day-level correlates of substance use. This may advance our understanding of the public health program needs related to substance use for populations who use crowdsourcing platforms in the United States.

In order to reduce this gap in research, we utilized daily dairies to identify the day-level correlates of substance use in a given day over a 2-week follow-up period among people who use alcohol or stimulants and who were recruited using MTurk in the United States. In addition, we assessed the relationship between key psychological factors measured at baseline and substance use in a given day over the 2-week follow-up period. More specifically, the main two objectives of this study were to (1) examine the relationship between day-level cravings for alcohol and/or stimulants (ie, crack cocaine, cocaine, or methamphetamine) and substance use in a given day, and (2) determine whether certain psychosocial factors, such as depression, negative affect, self-esteem, and stress measured at baseline, were associated with substance use in a given day over the follow-up period. We hypothesized that both day-level cravings for alcohol and stimulants, and psychosocial stressors would be key predictors of substance use among MTurk users. Taken together, this research aimed to identify the daily correlates and baseline predictors of substance use among people who use alcohol and/or stimulants and who were recruited using MTurk, which may help inform the development of interventions for populations who utilize crowdsourcing platforms in the United States.

## Methods

### Ethical Considerations

Baseline and follow-up data were drawn from the Stimulants and Alcohol use in MTurk Behavioral Assessments Study (“SAMBA”), a study designed to examine substance use and HIV-related sexual risk behaviors among men who have sex with men (MSM) and non-MSM who use alcohol and/or stimulants in the United States. All study procedures and materials were reviewed and approved by the Institutional Review Board at the University of California, San Francisco. All participants provided informed consent using an online consent form during the screening process.

### Recruitment, Screening, and Enrollment

A total of 272 participants in the United States were enrolled using MTurk between March 26 and April 13, 2018. Participants were recruited online using MTurk [23], which involved posting an initial “Human Intelligence Task” and then screening participants for eligibility. Participants were considered eligible if they (1) were at least 18 years old, (2) were able to speak English, and (3) reported alcohol or stimulant (ie, cocaine, crack cocaine, or methamphetamine) use in the past year at baseline. The SAMBA Study recruited for the following two eligible groups (1:1 ratio): MSM who use alcohol or stimulants and non-MSM who use alcohol or stimulants. The parent study was interested in examining both substance use and HIV-related risk behaviors, and explicitly sampled MSM because this population is disproportionately impacted by both substance use and HIV-related risk behaviors [24,25].

### Online Surveys

All surveys were administered online and completed using computers or smartphones. First, participants completed an initial survey to be screened for eligibility, and if they were considered eligible, they completed a baseline assessment followed by 14 daily surveys. Participants were compensated US $0.80 for completing the screener, US $5.00 for completing the baseline survey, and US $1.00 for completing each daily diary, and those who completed all 14 daily surveys received a US $6.00 bonus, resulting in a maximum of US $25.80 in compensation per participant. Research staff contacted participants through their individual MTurk accounts and provided a link to complete the assessments using a unique authenticator known as a single sign on token.

### Baseline Measures

#### Sociodemographics

We assessed sociodemographic factors, including age in years and race/ethnicity (White, Asian, African American/Black, Native American/Alaskan Native, and Hawaiian/Pacific Islander). Being a sexual minority male (ie, MSM) was assessed using a dichotomous measure created from the responses to the following questions: (1) “*What was your sex at birth (male/female)?*” and (2) “*Who do you have sex with (men/women/transgender females or transwomen/transgender males or transmen)?*” Data were also collected on relationship status (married/committed, single, and divorced), employment status (full time, part time, and unemployed), having health insurance (yes/no), ever testing for HIV (yes/no), and annual income (≥US $125,000, US $75,000-124,999, US $40,000-74,999, and ≤US $40,000). Having at least a 4-year degree was assessed by creating a dichotomous variable that included those who attained a bachelor’s degree or completed any postgraduate studies versus those who completed 12th grade/general education degree or an associate of arts degree/some college.

#### Alcohol Use

We measured alcohol consumption in the past 6 months (yes/no) and current or past hazardous drinking (yes/no) using the three-item Alcohol Use Disorders Identification Test-Concise (AUDIT-C), where scores ≥4 for males and ≥3 for females indicate hazardous drinking [26,27]. The AUDIT-C consists of three questions that are designed to help identify problematic alcohol use, and scores range from 0 to 12 (a score of 0 reflects no alcohol use in the past year) [26,27]. A higher AUDIT-C score represents a higher likelihood that the participant’s drinking is negatively affecting health [26,27].

#### Drug Use

Methamphetamine use, cocaine use, crack cocaine use, and any drug use, which included reporting any injection or noninjection drug use in the past 6 months (yes/no), were also assessed.

### Psychosocial Measures at Baseline

Self-esteem was assessed using the 10-item Rosenberg Self-Esteem Scale (RSES) [28,29], where higher scores represent higher levels of self-esteem (α=.92). Participants recorded their level of agreement with statements assessing general feelings related to esteem on a 4-point scale ranging from “strongly agree” to “strongly disagree.” Items 2, 5, 6, 8, and 9 were reverse coded to ensure that higher scores represent higher levels of self-esteem. Final scores were assessed by summing the scores from all 10 items.

Positive affect and negative affect were measured using the 20-item Positive and Negative Affect Schedule (PANAS) [30], where scores range from 10 to 50 and higher scores indicate higher levels of positive (α=.90) or negative (α=.94) affect. Using a 5-point scale (1=very slightly or not at all to 5=extremely), participants recorded their level of agreement with the 20 emotions assessed on the PANAS. Positive affect was measured by summing scores from items 1, 3, 5, 9, 10, 12, 14, 16, 17, and 19. Negative affect was measured by summing scores from items 2, 4, 6, 7, 8, 11, 13, 15, 18, and 20.

Stress was measured using the 10-item Perceived Stress Scale (PSS) [31], where scores range from 0 to 40 and higher scores represent higher levels of perceived stress. Using a 5-point scale (0=never to 4=very often), participants reported how often they experienced different feelings and thoughts in the past month. Items 4, 5, 7, and 8 were reverse coded to ensure that higher scores represent higher levels of stress. All items were summed to calculate total stress scores. For descriptive purposes, low stress (scores range from 0 to 13), moderate stress (scores range from 14 to 26), and high stress (scores range from 27 to 40) were also measured using the PSS.

Depression was measured using the 10-item Center for Epidemiologic Studies Depression Scale (CESD-10) [32], where scores range from 0 to 30 and scores ≥10 are considered to indicate depression (α=.91). Using a 4-point scale (0=rarely or none of the time/less than 1 day to 3=all of the time/5-7 days), participants reported how often in the past week they experienced different emotional states. Item 10 (“I could not get going”) was not included in the survey in error, so scoring for this item was performed by taking the average scores from items 1 to 9. Items 5 and 8 were reverse coded to ensure that higher scores represent higher levels of depression. All 10 items were summed to calculate total depression scores.

### Daily Diary Measures

#### Outcome

Our outcome of interest was a dichotomous measure of substance use in a given day that was created by combining heavy drinking and any drug use in the past 24 hours (yes/no). This measure included all individuals who reported heavy drinking or any drug use in the past 24 hours over the 14-day follow-up period (not at baseline). Heavy drinking in the past 24 hours (yes/no) was a dichotomous measure defined according to the National Institute on Alcohol Abuse and Alcoholism guidelines, which state that six or more drinks for males and five or more drinks for females per day can be considered heavy drinking [33]. Any drug use (including any injection and noninjection drug use) in the past 24 hours (yes/no) was a dichotomous measure derived from the following two questions: “In the past 24 hours, have you used any noninjection drugs recreationally or to get high (crystal meth/speed, crack or powder cocaine, marijuana, heroin, gamma-hydroxybutyric acid, prescription medications [such as oxycontin and xanax], hallucinogens [such as lysergic acid diethylamide], or others)?” and “Have you injected any drug in the past 24 hours?” All data for the outcome were collected after baseline via 14 daily diaries.

#### Cravings for Alcohol and Stimulants

In addition to alcohol and drug use, participants were asked to report their day-level cravings for alcohol and/or stimulants (ie, cocaine, crack cocaine, and methamphetamine) in the past 24 hours as appropriate. Craving scales ranged from 0 to 100, where 100 represents the strongest craving one has ever experienced and 0 represents no craving at all. Data on cravings were collected after baseline via 14 daily diaries.

### Statistical Analysis

Descriptive statistics were used to describe the study sample. For categorical variables, frequencies and percentages were used, and depending on distributional assumptions for continuous data (ie, normal distribution versus nonnormal distribution), means and SDs or medians and IQRs were used.

Logistic mixed effects regression models were used to analyze the associations between substance use and both time-invariant and time-varying factors over 14 days. Time-varying covariates were measured via 14 daily surveys, and day-level cravings for alcohol and stimulants (ie, cocaine, crack cocaine, and methamphetamine) were assessed. All models included a random intercept to account for repeated measures per person [34]. Measurements at timepoints for which there were no missing outcomes or covariate information were included in the model (there was a small amount of missing data, and this pattern is summarized below) [34].

We first built bivariate models to test each factor on substance use individually (a conservative Bonferroni multiple comparison correction for 24 tests would be *P*<.002). For those factors that were significantly (*P*<.051) associated with substance use, multivariable models were then built to determine if they independently contributed to substance use while controlling for potential confounders. Multivariable models were controlled for age and education because they have been identified as correlates of substance use in prior research [35,36]. Due to the high level of correlation among the psychosocial measures examined in this study (depression, affect, self-esteem, and stress), their effects on substance use were estimated in separate models, which also included the other exposures that were significant in bivariate analyses. A manual backward selection approach [37] that considered multicollinearity between all exposures was used to build all four final models. Variables that did not retain a *P* value that was <.051 were removed from the final models in order to achieve parsimony and enhance the fit of each model. The main effect of time over the follow-up was explored in all four models using an indicator variable for day of follow-up, but was not significant in any of the models and therefore was not included.

For all longitudinal data, including the outcome substance use in a given day and cravings for alcohol and stimulants in the past 24 hours, the overall percentage of missing data was calculated by summing the total number of missing responses and then dividing that number by the total number of potential responses (272×14=3808). The overall percentage of surveys completed was calculated by summing the number of surveys completed and dividing that number by the total number of surveys (3808). The average number of surveys completed per person was calculated by dividing the total number of surveys completed by the total sample size. All analyses were conducted using Stata 14.2 (Stata Corp).

## Results

### Screening, Enrollment, and Survey Completion Rates

A total of 3897 individuals responded to the MTurk task posted for this study. Of these, 2910 were screened out because they did not meet the eligibility criteria and another 41 were not included because they did not complete the screening survey. Of the 946 individuals who were considered eligible according to screening data, 161 were MSM and 785 were non-MSM. Of the 161 eligible MSM, 152 agreed to participate, and of the 785 eligible non-MSM, 781 agreed to participate, resulting in a total of 13 individuals who declined or opted out. However, this study only had the capacity to enroll 272 participants and had to waitlist the remaining 661. All of the participants who were enrolled consented at baseline. Completion rates for the 14 daily surveys were as follows: day 1, 99.3%; day 2, 98.2%; day 3, 99.3%; day 4, 95.9%; day 5, 94.1%; day 6, 91.9%; day 7, 95.9%; day 8, 95.2%; day 9, 95.9%; day 10, 94.8%; day 11, 92.6%; day 12, 90.8%; day 13, 92.3%; and day 14, 89.7%.

### Baseline Characteristics

Baseline characteristics of the study sample are described in Table 1. Among a total of 272 participants, 201 (73.9%) were male and 134 (65.3%) were MSM. The mean age was 36.1 years (SD 10.5). The majority of the sample identified as being White (220/272, 80.8%), followed by African American/Black (22/272, 8.0%), Asian (17/272, 6.2%), other (10/272, 3.6%), and Native American or Alaskan Native (3/272, 1.1%). Most participants were married or in a committed relationship (158/272, 58.0%). Less than half of the sample reported being single (112/272, 41.1%), and only 2 (0.7%) participants were divorced. Most participants (180/272, 66.1%) reported being fully employed, and over half (163/272, 59.9%) reported having at least a 4-year degree. Annual income varied, with slightly over a third (99/272, 36.4%) earning less than US $40,000, 89 (32.7%) earning US $40,000-74,999, 64 (23.5%) earning US $75,000-124,999, and 20 (7.3%) earning US $125,000 or more. The majority (229/272, 84.1%) of the sample reported having health insurance, and over half (170/272, 62.5%) reported ever being tested for HIV.

**Table 1:** Baseline sociodemographic characteristics, substance use, and psychosocial factors among people who use alcohol and/or stimulants recruited from MTurk between March 26 and April 13, 2018, in the United States (N=272).

Variable					Value
**Sociodemographic factors**					
	Age (years), mean (SD)			36.1 (10.4)	
	**Race/ethnicity, n (%)**				
		White	220 (80.8%)		
		Asian	17 (6.2%)		
		African American	22 (8.0%)		
		Native American or Alaskan Native	3 (1.1%)		
		Other	10 (3.6%)		
	**Gender/self-reported sex, n (%)**				
		Male	201 (73.9%)		
		Female	71 (26.1%)		
	Men reporting sex with other men, n (%)			134 (65.3%)	
	**Relationship status, n (%)**				
		Single	112 (41.1%)		
		Married/committed	158 (58.0%)		
		Divorced	2 (0.7%)		
	**Employment status, n (%)**				
		Full-time employment	180 (66.1%)		
		Part-time employment	45 (16.5%)		
		Unemployed	47 (17.2%)		
	Higher education (bachelor’s degree/any postgraduate studies), n (%)			163 (59.9%)	
	**Income (US $), n (%)**				
		≥125,000 (reference)	20 (7.3%)		
		75,000-124,999	64 (23.5%)		
		40,000-74,999	89 (32.7%)		
		<40,000	99 (36.4%)		
	Has health insurance, n (%)			229 (84.1%)	
	**Reported ever testing for HIV, n (%)**				
		Yes	170 (62.5%)		
		No	95 (34.9%)		
		Do not know	7 (2.5%)		
**Substance use**					
	Alcohol consumption in the past 6 months, n (%)			261 (99.2%)	
	AUDIT-C score, mean (SD)^a^			4.2 (2.4)	
	Hazardous alcohol consumption^b^, n (%)			173 (63.8%)	
	Methamphetamine use in the past 6 months, n (%)			19 (10.1%)	
	Cocaine use in the past 6 months, n (%)			31 (15.3%)	
	Crack cocaine use in the past 6 months, n (%)			8 (4.4%)	
	Any drug use in the past 6 months including injection drug use, n (%)			104 (38.2%)	
	**Substance use cravings^c^**				
		Day-level craving for alcohol in the past 24 hours, median (IQR)	5 (0-26)		
		Day-level craving for methamphetamine in the past 24 hours, median (IQR)	54 (20-88)		
		Day-level craving for cocaine in the past 24 hours, median (IQR)	39 (1-71)		
		Day-level craving for crack cocaine in the past 24 hours, median (IQR)	52 (51-87)		
**Psychosocial factors**					
	Self-esteem score^d^, median (IQR)			30 (26-35)	
	**Affect^e^**				
		Positive affect, median (IQR)	31 (26-37)		
		Negative affect, median (IQR)	15 (11-20)		
	**Perceived stress score^f^, median (IQR)**			17 (11-21)	
		Low stress, n (%)	93 (34.1%)		
		Moderate stress, n (%)	152 (55.8%)		
		High stress, n (%)	27 (9.9%)		
	Depression score^g^, median (IQR)			7.7 (3.3-12.2)	

^a^Alcohol Use Disorders Identification Test-Concise (AUDIT-C) scores were calculated using the three-item AUDIT-C.

^b^Hazardous drinking was measured at baseline using the three-item AUDIT-C. Scores range from 0 to 12. Scores of 4 or more for men indicate hazardous drinking and scores of 3 or more for women indicate hazardous drinking.

^c^Day-level craving scores range from 0 to 100.

^d^Self-esteem was measured using the “Rosenberg Self-Esteem Scale” (RSES). Higher scores represent higher self-esteem.

^e^Affect was measured using the “Positive and Negative Affect Schedule” (PANAS). Scores range from 10 to 50, with higher scores indicating higher levels of positive or negative affect. Positive and negative affect were measured by summing different items from the PANAS scale.

^f^Stress was measured using the “Perceived Stress Scale” (PSS). Scores range from 0 to 40, with higher scores representing higher perceived stress. Scores ranging from 0 to 13 are considered low stress, 14 to 26 are considered moderate stress, and 27 to 40 are considered high stress.

^g^Depression was measured using the 10-item Center for Epidemiologic Studies Depression Scale Revised (CESD-10). A score of ≥10 is considered to indicate depression.

Nearly all (261/272, 99.2%) participants reported consuming alcohol in the past 6 months. The mean AUDIT-C score for any current or past drinking was 4.2 (SD 2.4), and 173 (63.8%) participants engaged in any current or past hazardous alcohol consumption. In the past 6 months, 31 (15.3%) participants reported using cocaine, 19 (10.1%) reported using methamphetamine, 8 (4.4%) reported using crack cocaine, and 104 (38.2%) reported any noninjection or injection drug use. On a scale from 0 to 100, median day-level craving scores at baseline for alcohol, methamphetamine, cocaine, and crack cocaine were 5 (IQR 0-26), 54 (IQR 20-88), 39 (IQR 1-71), and 52 (IQR 51-87), respectively.

The median score for self-esteem was 30 (IQR 26-35). On a scale from 10 to 50, median scores for positive affect and negative affect were 31 (IQR 26-37) and 15 (IQR 11-20), respectively. On a scale from 0 to 40, the median perceived stress score was 17 (IQR 11-21), and just over half (152/272, 55.8%) of the sample reported experiencing moderate stress, followed by low stress (93/272, 34.1%) and high stress (27/272, 9.9%). On a scale from 0 to 30, where a score ≥10 is considered to indicate depression, the median score was 7.7 (IQR 3.3-12.2).

### Missing Data

Overall, there was a minimal amount of missing data. Out of a total of 3,808 possible responses, there were 201 (5.2%) missing responses for the primary outcome of interest (substance use in the past 24 hours over the follow-up period) and for day-level cravings for alcohol in the past 24 hours. With regard to day-level cravings for cocaine, crack cocaine, and methamphetamine in the past 24 hours, there were 253 (6.6%) missing responses for each measure of craving.

### Bivariate Analyses

Results from bivariate logistic regression models examining the predictors of substance use in a given day measured at baseline and each day over the follow-up period are summarized in Table 2, in addition to descriptive data stratified by substance use on day 1. Part-time employment was associated with a higher odds of substance use in a given day over the follow-up compared with full-time employment. Those who had a bachelor’s degree or completed some postgraduate work had a lower odds of engaging in substance use in a given day over the follow-up period compared with those who reported completing less education. Those who earned less than US $40,000 annually had a higher odds of substance use in a given day compared with those who earned US $125,000 or more annually. MSM had a higher odds of substance use in a given day compared with those who were not MSM.

**Table 2:** Bivariate random-intercept logistic regression models of the predictors of substance use in a given day among people who use alcohol and/or stimulants recruited from MTurk between March 26 and April 13, 2018, in the United States (N=272).

Variable			Substance use in the past 24 hours on day 1^a^ (n=50)	No substance use in the past 24 hours on day 1^a^ (n=220)	Unadjusted odds ratio (95% CI)	*P* value^b^
**Sociodemographic factors at baseline^c^**						
	Age (years), mean (SD)		35.50 (10.44)	36.32 (10.34)	0.99 (0.94-1.03)	.66
	**Race/ethnicity, n (%)**					
		White (reference)	40 (80.0%)	178 (80.9%)	N/A^d^	N/A
		Asian	1 (2.0%)	16 (7.3%)	0.19 (0.26-1.40)	.10
		African American	5 (10.0%)	17 (7.7%)	3.61 (0.62-21.05)	.15
		Native American or Alaskan Native	1 (2.0%)	2 (0.9%)	13.61 (0.10-1726.21)	.29
		Hawaiian or Pacific Islander	3 (6.00%)	7 (3.2%)	3.57 (0.28-44.84)	.32
	**Relationship status, n (%)**					
		Married/committed (reference)	28 (56.0%)	128 (58.2%)	N/A	N/A
		Single	22 (44.0%)	90 (40.9%)	1.53 (0.59-3.96)	.37
		Divorced	0 (0%)	2 (0.9%)	N/A	N/A
	**Employment status, n (%)**					
		Full-time employment (reference)	31 (62.0%)	149 (67.7%)	N/A	N/A
		Part-time employment	13 (26.0%)	32 (14.5%)	5.70 (1.54-21.00)	.009
		Unemployed	6 (12.0%)	39 (17.7%)	0.63 (0.18-2.18)	.46
	Higher education (bachelor’s degree or any postgraduate), n (%)		24 (48.0%)	138 (62.7%)	0.23 (0.08-0.59)	.003
	**Income (US $), n (%)**					
		≥125,000 (reference)	4 (8.0%)	16 (7.3%)	N/A	N/A
		75,000-124,999	3 (6.0%)	61 (27.7%)	0.39 (0.05-2.67)	.33
		40,000-74,999	14 (28.0%)	75 (34.1%)	1.03 (0.16-6.53)	.97
		<40,000	29 (58.0%)	68 (30.9%)	6.84 (1.09-42.81)	.04
	Has health insurance, n (%)		42 (84.0%)	185 (84.1%)	0.44 (0.12-1.64)	.22
	Ever tested for HIV, n (%)		36 (72.0%)	132 (60.0%)	0.69 (0.46-1.04)	.07
	Men reporting sex with other men, n (%)		37 (82.2%)	96 (60.8%)	7.35 (2.40-22.56)	<.001
**Substance use cravings at day one^e,f^**						
	Day-level craving for alcohol in the past 24 hours, median (IQR)		10.5 (0-50)	3 (0-15)	1.03 (1.02-1.04)	<.001
	Day-level craving for methamphetamine in the past 24 hours, median (IQR)		0 (0-9)	0 (0-0)	1.03 (1.01-1.05)	<.001
	Day-level craving for cocaine in the past 24 hours, median (IQR)		0 (0-1)	0 (0-0)	1.04 (1.02-1.06)	<.001
	Day-level craving for crack cocaine in the past 24 hours, median (IQR)		0 (0-0)	0 (0-0)	1.04 (1.02-1.06)	<.001
**Psychosocial factors at baseline^c^**						
	Self-esteem score^g^, median (IQR)		27.5 (23-32)	30 (27-36)	0.87 (0.81-0.93)	<.001
	Positive affect score^h^, median (IQR)		30 (25-34)	32 (26-38)	0.94 (0.89-1.00)	.07
	Negative affect score^h^, median (IQR)		19.5 (15-32)	14 (11-18.5)	1.14 (1.08-1.21)	<.001
	Perceived stress score^i^, median (IQR)		20 (15-23)	16 (10-20)	1.13 (1.06-1.20)	<.001
	Depression score^j^, median (IQR)		11.66 (4.44-17.77)	6.66 (2.22-12.22)	1.19 (1.11-1.27)	<.001

^a^In the bivariate logistic regression models, substance is the outcome and is a composite variable that includes those who reported heavy drinking and/or any drug use in the past 24 hours over a 2-week follow-up period. Substance use is the outcome and includes those who reported heavy drinking and/or any drug use in the past 24 hours on day 1.

^b^*P* values were derived from random effects logistic regression.

^c^Time invariant covariates measured at baseline.

^d^N/A: not applicable.

^e^Time varying covariates measured at day 1 over the follow-up period.

^f^Day-level cravings in the past 24 hours were assessed on a scale ranging from 0 to 100, where 0 is no craving at all and 100 is the strongest craving one has ever experienced.

^g^Self-esteem was measured using the “Rosenberg Self-Esteem Scale” (RSES). Higher scores represent higher self-esteem.

^h^Affect was measured using the “Positive and Negative Affect Schedule” (PANAS). Scores range from 10 to 50, with higher scores indicating higher levels of positive or negative affect. Positive and negative affect were measured by summing different items from the PANAS scale.

^i^Stress was measured using the “Perceived Stress Scale” (PSS). Scores range from 0 to 40, with higher scores representing higher perceived stress. Scores ranging from 0 to 13 are considered low stress, 14 to 26 are considered moderate stress, and 27 to 40 are considered high stress.

^j^Depression was measured using the 10-item Center for Epidemiologic Studies Depression Scale Revised (CESD-10). A score of ≥10 is considered to indicate depression.

Every 1-point increase in self-esteem was associated with a lower odds of engaging in substance use in a given day. Every 1-point increase in negative affect was associated with a higher odds of engaging in substance use in a given day. Similarly, increases in perceived stress and depression were associated with higher odds of engaging in substance use in a given day. Higher day-level craving scores in the past 24 hours for alcohol, cocaine, crack cocaine, and methamphetamine were all associated with a higher odds of engaging in substance use in a given day over the follow-up period.

### Multivariable Analyses

Results from all four multivariable logistic regression models examining baseline predictors and daily correlates of substance use in a given day while controlling for age in years and education are summarized in Table 3. In model 1, factors significantly associated with substance use in a given day were self-esteem (adjusted odds ratio [aOR] 0.90, 95% CI 0.82-0.98; *P*=.02), day-level craving scores for alcohol and cocaine (aOR 1.02, 95% CI 1.01-1.03; *P*<.001 and aOR 1.03, 95% CI 1.01-1.04; *P*=.01, respectively), and being in the MSM group (aOR 4.90, 95% CI 1.28-18.70; *P*=.02). In model 2, factors significantly associated with substance use in a given day were negative affect (aOR 1.08, 95% CI 1.01-1.16; *P*=.01), day-level craving scores for alcohol and cocaine (aOR 1.02, 95% CI 1.01-1.03; *P*<.001 and aOR 1.02, 95% CI 1.01-1.04; *P*=.001, respectively), and being in the MSM group (aOR 5.47, 95% CI 1.43-20.87; *P*=.01). In model 3, factors significantly associated with substance use in a given day were day-level craving scores for alcohol and cocaine (aOR 1.02, 95% CI 1.01-1.03; *P*<.001 and aOR 1.03, 95% CI 1.01-1.04; *P*=.001, respectively), and being in the MSM group (aOR 5.99, 95% CI 1.55-23.13; *P*=.009). In model 4, factors significantly associated with substance use in a given day were depression (aOR 1.11, 95% CI 1.02-1.21; *P*=.01), day-level craving scores for alcohol and cocaine (aOR 1.02, 95% CI 1.01-1.03; *P*<.001 and aOR 1.02, 95% CI 1.01-1.04; *P*=.001, respectively), and being in the MSM group (aOR 4.94, 95% CI 1.29-18.84; *P*=.01).

**Table 3:** Multivariable random-intercept logistic regression models of the predictors of substance use in a given day among people who use alcohol and/or stimulants recruited from MTurk between March 26 and April 13, 2018, in the United States (N=272).

Variable		Adjusted odds ratio (95% CI) Model 1^a,b^	*P* value^c^	Adjusted odds ratio (95% CI) Model 2^a,d^	*P* value^c^	Adjusted odds ratio (95% CI) Model 3^a,e^	*P* value^c^	Adjusted odds ratio (95% CI) Model 4^a,f^	*P* value^c^
**Sociodemographic factors measured at baseline^g^**									
	Mean age in years	1.01 (0.95-1.07)	.64	1.01 (0.95-1.07)	.62	1.01 (0.95-1.07)	.73	1.00 (0.95-1.06)	.78
	Higher education (bachelor’s degree/any postgraduate)	0.18 (0.05-0.61)	.006	0.17 (0.05-0.59)	.005	0.17 (0.05-0.60)	.006	0.19 (0.05-0.65)	.009
	Men reporting sex with other men	4.90 (1.28-18.70)	.02	5.47 (1.43-20.87)	.01	5.99 (1.55-23.13)	.009	4.94 (1.29-18.84)	.01
**Substance use day-level cravings measured over the follow-up^h,i,j^**									
	Day-level craving for alcohol in the past 24 hours	1.02 (1.01-1.03)	<.001	1.02 (1.01-1.03)	<.001	1.02 (1.01-1.03)	<.001	1.02 (1.01-1.03)	<.001
	Day-level craving for cocaine in the past 24 hours	1.03 (1.01-1.04)	.01	1.02 (1.01-1.04)	.001	1.03 (1.01-1.04)	.001	1.02 (1.01-1.04)	.001
**Psychosocial factors measured at baseline^g^**									
	Self-esteem score^k^	0.90 (0.82-0.98)	.02	N/A^l^	N/A	N/A	N/A	N/A	N/A
	Negative affect score^m^	N/A	N/A	1.08 (1.01-1.16)	.01	N/A	N/A	N/A	N/A
	Perceived stress score^n^	N/A	N/A	N/A	N/A	1.06 (0.98-1.16)	.11	N/A	N/A
	Depression score^o^	N/A	N/A	N/A	N/A	N/A	N/A	1.11 (1.02-1.21)	.01

^a^All adjusted models (1-4) are controlled for age in years and education (eg, having at least a BA degree).

^b^Total effect of self-esteem on substance use in a given day.

^c^*P* values were derived from random effects logistic regression.

^d^Total effect of negative affect on substance use in a given day.

^e^Total effect of perceived stress on substance use in a given day.

^f^Total effect of depression on substance use in a given day.

^g^Time invariant covariates measured at baseline.

^h^Time varying covariates measured at day 1 over the follow-up period.

^i^Day-level cravings in the past 24 hours were assessed on a scale ranging from 0 to 100, where 0 is no craving at all and 100 is the strongest craving one has ever experienced.

^j^Substance use includes those who reported heavy drinking and/or any drug use in the past 24 hours over a 2-week period.

^k^Self-esteem was measured using the “Rosenberg Self-Esteem Scale” (RSES). Higher scores represent higher self-esteem.

^l^N/A: not applicable.

^m^Negative affect was measured by summing certain items from the “Positive and Negative Affect Schedule” (PANAS).

^n^Stress was measured using the “Perceived Stress Scale” (PSS). Scores range from 0 to 40, with higher scores representing higher perceived stress.

^o^Depression was measured using the 10-item Center for Epidemiologic Studies Depression Scale Revised (CESD-10). A score of ≥10 is considered to indicate depression.

## Discussion

### Principal Findings

This daily diary study, which measured the predictors and day-level correlates of substance use in a given day among people who use alcohol and/or stimulants in the United States and who were recruited via MTurk, identified several important findings. Higher day-level craving scores for alcohol and stimulants predicted substance use in a given day over the 14-day follow-up period. Negative affect and depression measured at baseline were both associated with substance use in a given day. We also found that higher levels of self-esteem measured at baseline were associated with a lower odds of engaging in substance use in a given day over the follow-up period. These findings may have important implications for behavioral interventions that aim to reduce day-to-day patterns of heavy drinking and drug use among people who use alcohol and stimulants in the United States.

Using the daily dairy method, our study found day-level cravings for alcohol and stimulants to be correlated with substance use in a given day. Our study adds to existing research that supports the link between craving and substance use [15-17] by showing how cravings measured daily in one’s natural environment predict substance use among MTurk users in the United States. As such, results from this study may help further our understanding of how day-level fluctuations in cravings shape substance use patterns. Additionally, these findings point to the potential of the daily diary method in identifying high-risk days for substance use via monitoring cravings on a daily basis. This may be a promising opportunity to deploy mobile health (mHealth) or other technology-based interventions to address craving using empirically driven approaches [38]. For example, mHealth platforms, where participants can respond to drinking and drug use queries and receive timely feedback on how to avoid drug or alcohol use, have shown promise [39].

Negative affect was independently associated with substance use in a given day over the 2-week follow-up period in this study, which is consistent with prior research [40-43]. Based on this finding and former research, we believe that behavioral interventions should consider utilizing strategies to promote emotional regulation through enhancing positive emotion and sensitizing individuals to natural rewards [44]. Future studies should test whether an increase in positive affect leads to an increase in emotional regulation and a decrease in substance use. Further, utilizing technology-based interventions that address substance use in real-time and leverage mobile platforms or computers to deploy interventions may increase accessibility to efficacious treatments for people with SUD [45]. Technology-based interventions have also been proven to be cost-effective and thus a practical option in resource-limited settings [46].

Depression was associated with substance use in a given day in this study. Individuals who experience depression and engage in substance use tend to have worse treatment outcomes for both depression and substance use compared to those who experience one of these conditions alone [47]. Moreover, depression accelerates the onset of SUD and predicts relapse among people who use drugs [48]. Thus, it is recommended to treat the underlying mechanisms of both depression and substance use using transdiagnostic approaches that integrate treatments for both disorders [47]. For instance, cognitive behavioral therapy, mindfulness mediation, and acceptance-based approaches have all shown promise in addressing both depression and substance use [47], and should be considered in future intervention work. Moreover, to best address the needs of populations who use crowdsourcing platforms, delivering combined therapies that simultaneously address depression and substance use using technology-based approaches may be beneficial [38,39,49].

Higher levels of self-esteem were associated with a reduced odds of engaging in substance use over the follow-up period in our study. Previous studies have shown that self-esteem is protective against substance use and mediated by adaptive coping mechanisms among multiracial youth and college students in the United States [12,13]. Our study adds to this literature [12,13] by showing that higher levels of self-esteem are protective against substance use among MTurk-recruited adults who use alcohol and stimulants in the United States. Based on our findings and prior research [12,13], interventions for substance use should consider leveraging evidenced-based techniques, such as cognitive behavioral therapy and motivational interviewing, to enhance self-esteem and increase adaptive coping skills using technology-based platforms to engage larger populations of MTurk users [46].

MSM had significantly higher odds of engaging in substance use in a given day over the follow-up period compared to those who were not MSM in our study. Drug and alcohol use are common among MSM and have been linked to chronic stress due to sexual stigma, depression, sexual anxiety, gay community attachment, and internalized homophobia [50]. MSM also report using drugs and alcohol to enhance their sense of belonging, help cope with everyday life stress, and increase their sense of pleasure [51]. In order to reduce substance use among MSM, it is imperative to develop culturally appropriate substance use treatment programs [52] that take the underlying drivers of substance use specific to MSM into account [50].

Perceived stress was not independently associated with substance use in a given day in our study. One possible explanation for this is that our baseline measure of stress was not collected close enough to the repeated measures outcome of interest to detect an association. Stress may be a more transient experience that should be captured using repeated measures data. We recommend that future studies leveraging a repeated measures deign collect data on stress closer to the outcome measure of substance use to better understand the potential temporal effect of stress on substance use. Further, since the relationship between stress and substance use has been established in other studies [10,11], we recommend interpreting our findings with caution and continuing to address stress by enhancing adaptive coping mechanisms in interventions for substance use.

### Limitations

This study has limitations. We relied on self-reported data of sensitive behaviors, such as drug and alcohol use, that were collected via daily diaries, which may be subject to social desirability bias and recall bias. These biases may threaten the reliability and validity of our findings by dampening the effect or pushing the results toward the null. However, it should be noted that the daily diary method is known to enhance the ecological validity of substance use research by collecting repeated measures over time in one’s natural environment [17]. The lack of racial and ethnic diversity in our sample may limit the generalizability of our results to other populations of people who use alcohol and/or stimulants. Future MTurk studies should develop strategies to recruit more diverse samples of people who use alcohol and stimulants to broaden the applicability of the research findings [53,54]. Aside from MSM, we were not powered to detect any potential relationship between other sexual minority groups, including women who have sex, and substance use, which may limit the generalizability of our findings to sexual minorities other than MSM. All psychosocial measures were collected at baseline only; therefore, no time-varying effect can be inferred from the detected associations. Further, the relationship between stress and substance use may not have been detected because stress was not measured close enough to the repeated measures outcome. Item 10 of the CESD-10 (“I could not get going”) was not included in the study survey in error, so scoring for this item was performed by taking the average scores from items 1 to 9, which may compromise the validity of this item. Regression analyses were performed using data for complete cases only (ie, all cases with missing outcome or covariate data were excluded). Complete case analysis assumes that data are missing completely at random, which means that the cause of missing data is independent of the observed (ie, the measured outcome of interest) and unobserved (ie, other unmeasured causes) parameters of interest [34]. Although this is a strong assumption, it should be noted that the largest overall percentage of missing responses was very minimal (6.6%, for day-level cravings for stimulants in the past 24 hours) and approximately meets the rule of thumb for such an analysis [55]. Despite these limitations, this study provides several important insights into the predictors and day-level correlates of substance use among people who use alcohol and/or stimulants in the United States and who were recruited via MTurk.

In summary, day-level cravings for alcohol and stimulants, depression, negative affect, and being in the MSM group predicted substance use, and higher levels of self-esteem were protective against substance use in our sample of people who used alcohol and/or stimulants. Interventions that target biobehavioral circuitries, such as craving, should be investigated in conjunction with programs that are designed to reduce negative psychosocial stressors like depression. Substance use treatment programs may also consider employing cognitive behavioral strategies to enhance self-esteem and improve adaptive coping. Further, culturally tailored approaches should be developed to effectively engage MSM in interventions. Finally, we recommend delivering interventions for substance use using mHealth or other technology-based platforms, in order to increase the accessibility to efficacious treatments for people living with SUD in the United States.

## Abbreviations

aOR: adjusted odds ratio
AUDIT-C: Alcohol Use Disorders Identification Test-Concise
CESD-10: Center for Epidemiologic Studies Depression Scale
mHealth: mobile health
MSM: men who have sex with men
MTurk: Amazon Mechanical Turk
PANAS: Positive and Negative Affect Schedule
PSS: Perceived Stress Scale
SAMBA: Stimulants and Alcohol use in MTurk Behavioral Assessments Study
SUD: substance use disorder
